# *In Vivo* Capsular Switch in *Streptococcus pneumoniae* – Analysis by Whole Genome Sequencing

**DOI:** 10.1371/journal.pone.0047983

**Published:** 2012-11-08

**Authors:** Fen Z. Hu, Rory Eutsey, Azad Ahmed, Nelson Frazao, Evan Powell, N. Luisa Hiller, Todd Hillman, Farrel J. Buchinsky, Robert Boissy, Benjamin Janto, Jennifer Kress-Bennett, Mark Longwell, Suzanne Ezzo, J. Christopher Post, Mirjana Nesin, Alexander Tomasz, Garth D. Ehrlich

**Affiliations:** 1 Center for Genomic Sciences, Allegheny Singer Research Institute, Pittsburgh, Pennsylvania, United States of America; 2 Department of Microbiology and Immunology, Drexel College of Medicine, Pittsburgh, Pennsylvania, United States of America; 3 Deparment of Otolaryngology Head and Neck Surgery, Drexel College of Medicine, Pittsburgh, Pennsylvania, United States of America; 4 Laboratory of Molecular Genetics, Instituto de Tecnologia Química e Biológica Oeiras, Portugal; 5 Laboratory of Microbiology and Infectious Diseases, Rockefeller University, New York, New York, United States of America; Instituto Butantan, Brazil

## Abstract

Two multidrug resistant strains of *Streptococcus pneumoniae* – SV35-T23 (capsular type 23F) and SV36-T3 (capsular type 3) were recovered from the nasopharynx of two adult patients during an outbreak of pneumococcal disease in a New York hospital in 1996. Both strains belonged to the pandemic lineage PMEN1 but they differed strikingly in virulence when tested in the mouse model of IP infection: as few as 1000 CFU of SV36 killed all mice within 24 hours after inoculation while SV35-T23 was avirulent.

Whole genome sequencing (WGS) of the two isolates was performed (i) to test if these two isolates belonging to the same clonal type and recovered from an identical epidemiological scenario only differed in their capsular genes? and (ii) to test if the vast difference in virulence between the strains was mostly – or exclusively – due to the type III capsule. WGS demonstrated extensive differences between the two isolates including over 2500 single nucleotide polymorphisms in core genes and also differences in 36 genetic determinants: 25 of which were unique to SV35-T23 and 11 unique to strain SV36-T3. Nineteen of these differences were capsular genes and 9 bacteriocin genes.

Using genetic transformation in the laboratory, the capsular region of SV35-T23 was replaced by the type 3 capsular genes from SV36-T3 to generate the recombinant SV35-T3* which was as virulent as the parental strain SV36-T3* in the murine model and the type 3 capsule was the major virulence factor in the chinchilla model as well. On the other hand, a careful comparison of strains SV36-T3 and the laboratory constructed SV35-T3* in the chinchilla model suggested that some additional determinants present in SV36 but not in the laboratory recombinant may also contribute to the progression of middle ear disease. The nature of this determinants remains to be identified.

## Introduction

*Streptococcus pneumoniae* is a naturally transformable gram positive bacterium that is normally found in the human nasopharynx and its contiguous anatomic structures [1], [2]. A recent study in European Day Care Centers reported that over 95% of children were colonized by pneumococci at least once during the study [3] and in developed and developing countries alike, virtually every child becomes a nasopharyngeal carrier of *S. pneumoniae* during the first year of life [4]. Although *S. pneumoniae* commensally colonizes healthy children and adults, it has also remained an opportunistic pathogen even after the introduction of antibiotics into the environment and *S. pneumoniae* infections are associated with over 1 million deaths worldwide on an annual basis, mostly in children under the age of 5 who develop invasive sepsis and meningitis [5]. Pneumococcus has also remained a leading cause of community acquired pneumonia among the elderly and immune-compromised in Europe and the USA, and it accounts for approximately 30% of all hospitalized cases of community pneumonia which has a case fatality rate of 10–30% [6], [7].

Most of the disease burden associated with the pneumococcus results from mucosal infections such as sinusitis and otitis media (OM) [4]. OM is responsible for approximately 5 billion dollars a year in health care costs in the United States [8] and is the number one disease for which children visit a physician, receive antibiotic therapy and undergo surgery with a general anesthetic in the USA [9]. Pneumococcus is the most frequent organism isolated from OM effusions both acute and chronic [8]–[10].

Studies in animal models have identified numerous components of *S. pneumoniae* that contribute to the virulence of this pathogen (for review see [11]). The most important of these virulence factors is the capsular polysaccharide which protects the bacteria against the host immune system. Strains of *S. pneumoniae* are capable of producing as many as 93 capsular polysaccharides of different chemical structure [12], [13] and a retrospective meta-analysis has identified the capsular polysaccharide of pneumococcus as a major and independent determinant of human invasive pneumococcal disease [14]. When human isolates of *S. pneumoniae* were tested in mouse models a strong association was found between capsular type and virulence [15].

An important feature of *S. pneumoniae* is its ability to undergo genetic transformation by taking up and recombining with homologous and/or heterologous DNA molecules and thus acquire genetic traits that enable the bacteria to survive and cope with such powerful selective pressures as antibiotics and vaccines. The importance of this genetic mechanism for the evolution of *S. pneumoniae* in the *in vivo* environment has been documented by the whole genome sequencing of *S. pneumoniae* isolates belonging to the PMEN1 clone, one of the major multidrug resistant lineages that spread extensively through the globe [16]. Multiple genetic exchanges have also been demonstrated in a recent study of a polyclonal infection in a pediatric patient and these multiple gene acquisition events may have contributed to the prolonged course of disease [17]. It is most likely that such genetic events take place in the ecological reservoir of *S. pneumoniae* – the human nasopharynx – which was shown to be inhabited by multiple clonal types of pneumococci [18].

Recombinational events that lead to the acquisition of a new capsular type are of particular importance since they can profoundly alter the virulence potential of the bacteria and in the study described here we report on the analysis of such an *in vivo* capsular switch event which was identified during an outbreak investigation of pneumococcal disease at a health care facility in New York [19], [20]. All *S. pneumoniae* isolates – including the two strains analyzed here – were recovered from various body sites of patients during the outbreak investigation [20] and molecular typing indicated that they all belonged to the multidrug resistant PMEN1 clone of *S. pneumoniae* which most frequently produces a capsular 23F polysaccharide. On the other hand, a few of the PMEN1 isolates expressed capsular type 3 which is rare in this pneumococcal lineage. Comparison of one of these type 3 isolates SV36-T3 with one of the 23F strains SV35-T23 in the mouse model of intra-peritoneal infection showed that the capsular type 3 isolate was highly virulent: as few as 1000 colony forming units (CFU) of the bacteria were able to kill all experimental animals within less than a day. In contrast, the isolate with the 23F capsule was virtually avirulent [21].

In the study described here, we used whole genome sequencing in order to compare in more detail the genetic makeup of isolates SV35-T23 and SV36-T3 and also to identify the primary genetic determinant(s) responsible for the very large increase in the virulence potential of the capsular type 3 isolate.

## Materials and Methods

### Ethics statement

All animal experiments were conducted with either the approval of the Allegheny Singer Research Institute (ASRI) or the Rockefeller University Institutional Animal Care and Use Committee (IACUC).

### Culture of bacterial strains

The PMEN1 clinical *S. pneumoniae* strains SV35-T23 (capsular type 23) and SV36-T3 (capsular type 3), and the laboratory-constructed strains SV35-T3* and SV36-T3* were all cultured in either Columbia broth and Columbia agar or Todd Hewitt broth (THB) and Todd Hewitt agar at 37°C with 5% carbon dioxide supplementation. Antibiotics were added as appropriate. All broth cultures were incubated without shaking.

### Whole genome sequencing of the two clinical isolates: SV35-T23 and SV36-T3

We performed high coverage (>25×) 454-based whole genome sequence (WGS) analysis of the strains using the FLX-Titanium platform, and the contig data was deposited at GenBank under accession numbers ADNN for SV35-T23 and ADNO for SV36-T3 [22]. Detailed comparative genomic analyses was performed using MAUVE [17], [23] and DEP3 [24], [25]. Whole genome sequences of SV35-T23 and SV36-T3 were aligned against reference sequence ATCC700669 using MAUVE [23]. Locations from this alignment matching the 11 neighbor groups were visualized in CIRCOS [26].

### Capsule switch methods

#### Construction of strain SV35-T3*

Expressing a type III capsule – involved a two-step procedure.

Step 1 was the introduction of a spectinomycin resistance marker into the capsular region of SV36-T3. In order to “equip” strain SV36-T3 with a selectable marker in the capsule region, the transposon (SV36-T3_312) from the SV36-T3 capsule region and approximately 1500 bases upstream and downstream were amplified by PCR using primers 12921/12922 (**Table S1**). This PCR product was then ligated into the plasmid pGem-T Easy and transformed by electroporation into Top 10 *E. coli*. Transformants were selected on LB agar impregnated with ampicillin (100 µg/ml) after overnight incubation at 37°C. Transformants were used for preparation of mini plasmids which were tested by PCR for the correct insertion using primers 12921/12922. Inverse PCR was done on the plasmid (pGem-T tnp1) using primers 12956/12957 to create an open plasmid with restriction sites in the middle of the transposon. These primers were designed to have *Xma* I and *Bam HI* restriction sites in their 5′ termini, respectively, to facilitate cloning. The PCR yielded a linearized plasmid with restriction sites on each end. A spectinomycin resistance cassette was amplified from plasmid pR412 (a gift from Donald Morrison) using the primers 13021/13022. Plamid pR412 also contains the restriction sites *Xma I* and *Bam HI*. Both of these PCR products were cut with their respective restriction enzymes to generate sticky ends. They were then ligated together and transformed into Top 10 *E. coli* by electroporation. Transformants were selected on LB spectinomycin (100 µg/ml) plates. The resulting plasmid, pGem-T_ tnp1_Spec, was confirmed by PCR using primers12921/12922 and 13021/13022. This plasmid which has the spectinomycin cassette in the middle of the transposon was then linearized by digestion with *Sac I*. The linearized plasmid was next transformed into *S. pneumoniae* SV36-T3 as follows. Cultures were grown to OD_600_ = 0.05 followed by mixing 2 mL of the culture with 100 µL bovine serum albumin (4%), 10 µL CaCl_2_ (1%), 10 µL competence stimulating peptide 2 (20 µg/ml) (a gift of Donald Morrison), and 2 µg linearized transforming DNA. This mixture was incubated at 37°C, 5% CO_2_, for 1 hr. After incubation, 4 layer plates were made out of the transformation as follows: the first layer was composed of 3 mL of Columbia agar; the second layer was composed of 2 mL Columbia agar plus 1 mL of the transformation mixture; the third layer was composed of 3 mL Columbia agar; and the fourth layer was composed of 3 mL of Columbia agar+spectinomycin (400 µg/ml)−final concentration = 100 µg/ml for the entire plate). Plates were incubated overnight at 37°C with 5% CO_2_. Transformants were grown up in Columbia broth supplemented with spectinomycin (100 µg/ml), and were confirmed by PCR using primers 12921/12922 and 13021/13022.

**Figure 1:**
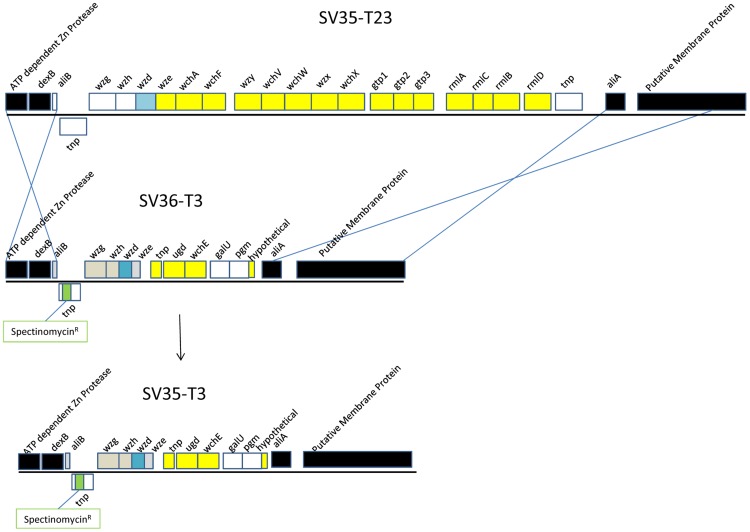
Annotation of the capsular biosynthetic gene regions of SV35-T23 and SV36-T3. Whole genome sequencing using the 454 Life Sciences Titanium platform was followed by automated annotation and manual curation of all gene possession differences between SV35-T23 and SV36-T3 (gray: pseudogenes, blue: orthologous genes, yellow: unique genes, black: flanking genes; green: spectinomycin resistance cassette; white: genes present in both genomes and functional in at least one). These data were used to identify and characterize the type 23 and type 3 capsular biosynthetic genes, as well as to identify the contiguous regions of identity between the two strains. The panel shows a diagram of the capsular switch strategy highlighting the movement of the SV36-T3 capsular region containing the inserted spectinomycin-resistance cassette into the SV35-T23 where it replaces the type 23 capsule genes.

Step 2 was the construction of SV35-T3* by genetic transformation.From the SV36-T3_tnp1_Spec construct – named SV36-T3* - the capsule region containing the spectinomycin cassette as well as 4000 bases up and downstream of the capsule region, (sequences which are identical in strain SV35-23), were amplified by PCR using primers 13044/13045 and this PCR product was then used to transform competent cells of SV35-T23. The SV35-T3* transformants were selected on plates containing spectinomycin. Included at the 3′ end of the 22 kb amplimer are genes/pseudogenes which are sometimes considered part of the type 3 capsule and sometimes not (these include: glucose 1-phosphate uridylyltransferase (*galU*), phosphoglucomutase (*pgm*), and a partial coding sequence (white in the schematic of SV36-T3 and SV35-T3* in **Figure 1**). The *galU* and *pgm* genes encode enzymatic functions used in the production of the type 3 capsule, but are not essential since there exist core cellular genes in the pneumococcus that encode the same functions.

**Figure 2:**
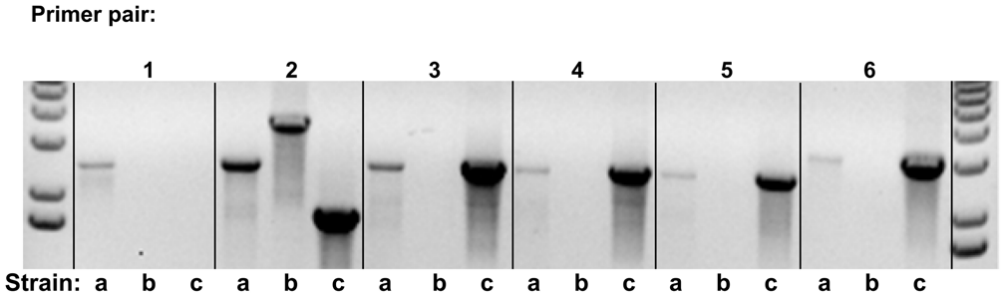
PCR confirmation of capsule-switch in SV35-T23 producing SV35-T3. Six sets of primers (with one exception) were designed from the SV36-T3 genome to produce products that had small overlaps with the adjacent amplimers to ensure complete coverage of the entire capsular region. Diagnostic amplifications were done with each of the primer sets for each of the three pneumococcal strains: SV35Type3 spec (SV35-T3) (a), SV35-T23 (b), and SV36-T3 (c) from left to right. Primer pair #1 included a reverse primer in the spectinomycin resistance cassette explaining the missing SV36-T3 genomic DNA band. Primer pair #2 shows the presence of the spectinomycin resistance cassette in SV35-T3 which produces a larger band relative to SV36-T3. The SV35-T23 genomic DNA also produces a product, larger in size than the type 3 strains, because of homology between the capsular regions and the fact that the type 23 capsule region includes many more genes and is therefore larger. Primer pairs #3–6 show type 3 capsular DNA bands in the recombinant SV35-T3 strain that are identical in size to the SV36-T3 parental strain and which are absent in the SV35-T23 (type 23 capsule) genomic DNA.

The serotype of the recombinant strain SV35-T3* was confirmed by the Quellung reaction, by its mucoid colony appearance characteristic of the type 3 capsule, and by diagnostic PCR amplifications using primers 13106/13107, 13108/13109, 13110/13111, 13112/13113, 13114/13115, 13116/13117 (**Figure 2**).

#### Murine Intraperitoneal Infection Model

Groups of 8-week-old female CD1 outbred mice were obtained from the Charles River Laboratories (Wilmington, MA). During the experimental period all mice were monitored on a daily basis for survival. An intraperitoneal (i.p.) injection of 75 µl of a xylazine and ketamine mixture was used to anesthetize the animals. CFU numbers inoculated into the mice were confirmed by colony count of serial dilutions on TSA plates supplemented with 6% sheep's blood (and 5 µg/ml gentamycin when appropriate). Mice surviving the infections for 7 days after the i.p. inoculation were euthanized by injection of 100 µl pentobarbital sodium (Nembutal) into the peritoneal space. Mice were given food and water *ad libitum*. Groups of mice (5 for each bacterial inoculum and bacterial concentration) were injected intraperitoneally with 500 µl of inoculum containing 10^3^ CFU (strains SV36-T3, SV35-T23 and the two strains carrying the spectinomycin resistance marker: SV36-T3* and SV35-T3*). The rate of survival of the animals was evaluated at various time points (days 1, 2, 3, etc.) after inoculation. In addition, because there was no mortality associated with the SV35-T23 strain, an additional experiment was run using 10^7^ CFU of this strain.

#### Chinchilla model of otitis media

All chinchilla experiments were conducted with the approval of the Allegheny Singer Research Institute (ASRI) Institutional Animal Care and Use Committee (IACUC). Research grade young adult chinchillas (*Chinchilla laniger*) weighing 400–600 gm (McClenahan Chinchilla Ranch, New Wilmington, PA) were obtained free of middle-ear disease as determined by otoscopy. After a protocol-directed period of environmental acclimation, induction of anesthesia was attained on day 0 by intramuscular injection of 0.1 mL of a solution of ketamine hydrochloride 100 mg/mL, xylazine hydrochloride 30 mg/mL and acepromazine 5 mg/mL. After anesthesia was confirmed (abolishment of the eye-blink reflex), 0.1 mL of bacterial cultures containing 100 CFU was injected bilaterally into the tympanic bullae using a 0.5 in, 27-gauge needle attached to a 1 mL syringe. All cultures were titered by plate dilution following inoculation and were within 25% of the target CFU numbers. Each of the four pneumococcal strains – SV35-T23, SV36-T3 and SV36-T3* and SV35-T3* (strains carrying a spectinomycin resistance marker) –were used to infect cohorts of at least 9 chinchillas. A multi-parameter scoring system based on signs of local and systemic disease [27], [28] was applied to each chinchilla on a daily basis for ten days to assess the status of the tympanic membrane and the progression and degree of otoscopic and systemic disease induced by the various pneumococcal strains. All clinical evaluations were performed by a board-certified otolaryngologist who was blinded with respect to the strains used to inoculate the animal. Moribund animals were sacrificed in accordance with the IACUC protocol. Once an animal reached this state, evaluation ceased, as moribundity was considered an end point.

## Results and Discussion

Between the end of December 1995 and January 1996, five individuals residing in the AIDS care unit of the long-term care facility of St. Vincent's Hospital, New York developed *S. pneumoniae* disease – pneumonia and/or bacteremia and two patients with bacteremic disease died. Investigation of the outbreak identified the *S. pneumoniae* strain responsible and sputum samples showed that the strain had also colonized other residents of the AIDS care unit. The strain was resistant to multiple drugs including penicillin, cefotaxim, erythromycin, tetracycline, rifampin and trimethoprim-sulfa-methoxezole and application of molecular typing (PFGE) indicated that the strain involved belonged to the pandemic clone PMEN1 [19], [20]. While most isolates expressed capsular polysaccharide 23F – characteristic of this *S. pneumoniae* lineage – a few of the isolates – including SV36 – produced a type 3 capsule instead. Nevertheless, both capsular type 23F and type 3 strains showed a common sequence type MLST 81, identical PFGE profile and identical restriction pattern of the *PBP1A, 2x and 2B* genes [21].

The identical clonal type of SV35-T23 and SV36-T3 and the epidemiological scenario from which they were recovered suggested that they represented a case of an *in vivo* capsular switch in which a representative of the penicillin resistant PMEN-lineage – SV35-T23 – has replaced its capsular gene complex 23F – typical of this lineage – by determinants of the capsular polysaccharide 3, thus generating the *in vivo* transformant SV36-T3 which may only differ from strain SV35-T23 in its different capsular gene complex. While the nature of the *in vivo* donor of the type 3 capsular genes remains unknown, the consequence of these genetic events for the virulence potential of this strain was profound: as many as 10^7^ CFU of SV35-T23 allowed survival for over 7 days of all mice intraperitoneally inoculated with this strain. In contrast as few as 10^3^ CFU of strain SV36-T3 killed all experimental animals within 24 hours.

The purpose of experiments described in this communication was two-fold: (i) to compare the genetic makeup of the two PMEN1 strains – SV35-T23 and SV36-T3 – with higher resolution and (ii) to test whether or not the enormous difference between the virulence of the two strains was due exclusively to the difference between capsular types.

In order to test this, we performed whole genome sequencing of the two isolates with a 454-based whole genome sequence (WGS) analysis using the FLX-Titanium platform.

### Genetic differences between strains SV35-T23 and SV36-T3

Detailed comparative genomic analyses demonstrated that, although highly related the two strains SV35-T23 and SV36-T3 did vary at multiple loci both in terms of their possession of distributed genes and also having different allelic forms of a small subset of core genes.

**Figure 3:**
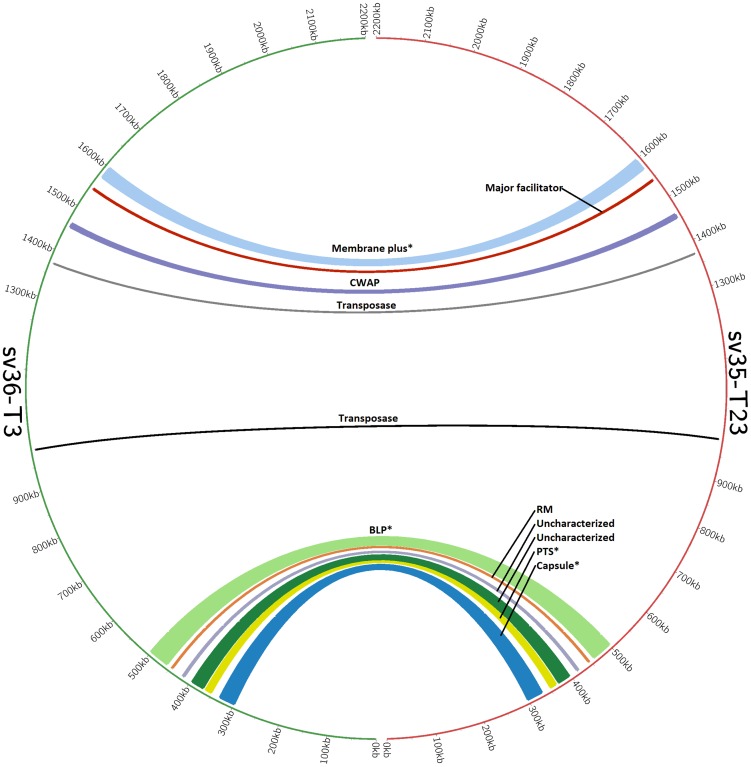
Visualization of neighbor groups between strains SV35-T23 and SV36-T3 using the CIRCOS representation tool. Each one of the 11 arches represents a neighboring group, the numbering around the circle indicates the position on the chromosome, and the width of the arch correlates to the size of the variant region. ‘*’mark the four regions that contain genic differences in addition to allelic differences. Regions are named relative to a coding sequence of interest, and abbreviations are as follows: restriction modification system (RM), cell wall associated protein (CWAP), phosphotransferase system (PTS), and bacteriocin locus (BLP).

We identified eleven “neighbor groups”: contiguous genomic regions, which differed between SV35-T23 and SV36-T3 and these genomic regions are represented by the arches in Figure 3. Each arch in Figure 3 identifies the location of a single neighbor group and has scales that correspond to the size of the neighbor group. Colors are arbitrary and used only to differentiate between locations.

The eleven groups are composed of approximately 158 kilobases of sequence (Kb) in SV35-T23 and approximately 135 Kb in SV36-T3. They contained a total of about 2500 single nucleotide polymorphisms – indicating that the two strains that were both members of the same PMEN1 clone and were recovered from the same outbreak nevertheless had significant differences in their evolutionary history. The nature of selected pressures responsible for the recruitment of such strains into a common epidemic setting is unknown.

Seven of the eleven neighbor groups consisted of allelic forms of genes which are core for the two strains. Four of the eleven neighbor groups contained 36 genetic determinants that were unique to the two strains, i.e., they were present in only one of the strains (these non-core regions are marked with ‘*’ in Figure 3). Twenty five of these genes are unique to SV35-T23 and include 15 genes that encode the biosynthesis of the type 23F polysaccharide, 5 genes of phosphotransferase system and 5 additional determinants that include bacteriocin genes.

In the case of SV36-T3, the 11 unique determinants include four genes that encode the biosynthesis of the capsular type 3 polysaccharide, determinants of an integrase domain and some bacteriocin loci (see Tables 1, 2, 3).

**Table 1:** Genes Unique to Strain SV35-T23.

Functional Category	Annotation	SV35-T23 ID
type 23 capsular locus	capsule biosynthesis tyrosine-protein kinase - **wze**	CGSSpSV35_0503
type 23 capsular locus	undecaprenylphosphate glucosephosphotransferase (initial sugar transferase) - **wchA**	CGSSpSV35_0504
type 23 capsular locus	putative rhamnosyl transferase - **wchF**	CGSSpSV35_0505
type 23 capsular locus	oligosaccharide repeat unit polymerase - **wzy**	CGSSpSV35_0506
type 23 capsular locus	putative glycosyltransferase - **wchV**	CGSSpSV35_0507
type 23 capsular locus	putative glycosyltransferase - **wchW**	CGSSpSV35_0508
type 23 capsular locus	capsule biosynthesis repeating unit flippase - **wzx**	CGSSpSV35_0509
type 23 capsular locus	putative glycerol phosphotransferase - **wchX**	CGSSpSV35_0510
type 23 capsular locus	putative glycerol-2-phosphate dehydrogenase - **gtp1**	CGSSpSV35_0511
type 23 capsular locus	putative nucleotidyl transferase - **gtp2**	CGSSpSV35_0512
type 23 capsular locus	putative phosphotransferase - **gtp3**	CGSSpSV35_0513
type 23 capsular locus	glucose-1-phosphate thymidyl transferase - **rmlA**	CGSSpSV35_0514
type 23 capsular locus	dTDP-4-keto-6-deoxyglucose-3,5-epimerase - **rmlC**	CGSSpSV35_0515
type 23 capsular locus	dTDP-glucose-4,6-dehydratase - **rmlB**	CGSSpSV35_0516
type 23 capsular locus	dTDP-4-dehydrorhamnose reductase - **rmlD**	CGSSpSV35_0517
phosphotransferase system (PTS)	putative mannitol-specific phosphotransferase system (PTS), IIBC component	CGSSpSV35_0553
phosphotransferase system (PTS)	putative transcriptional regulator	CGSSpSV35_0554
phosphotransferase system (PTS)	putative mannitol-specific phosphotransferase system (PTS), IIA component	CGSSpSV35_0555
phosphotransferase system (PTS)	mannitol-1-phosphate 5-dehydrogenase	CGSSpSV35_0556
phosphotransferase system (PTS)	ABC-type polar amino acid transport system, ATPase component	CGSSpSV35_0557
bacteriocin locus	pncR	CGSSpSV35_0662
bacteriocin locus	pncT	CGSSpSV35_0663
bacteriocin locus	bacteriocin blpM (or pncI)*	CGSSpSV35_0665
	hypothetical protein	CGSSpSV35_1729
	hypothetical protein	CGSSpSV35_1730

**Table 2:** Genes Unique to Strain SV36-T3.

Functional Category	Annotation	SV36-T3 ID
type 3 capsular locus	transposase domain protein - **tnp**	CGSSpSV36_0284
type 3 capsular locus	UDP-glucose 6-dehydrogenase (UDP-Glc dehydrogenase)(UDP-GlcDH) (UDPGDH) - **ugd**	CGSSpSV36_0285
type 3 capsular locus	glycosyl transferase family 2 family protein - **wchE**	CGSSpSV36_0286
type 3 capsular locus	hypothetical protein	CGSSpSV36_0289
Integrase where PTS exists in SV35-T23	integrase. domain protein	CGSSpSV36_0316
bacteriocin locus	putative bacteriocin BlpI	CGSSpSV36_0025
bacteriocin locus	putative bacteriocin BlpJ	CGSSpSV36_0026
bacteriocin locus	putative bacteriocin BlpK	CGSSpSV36_0027
bacteriocin locus	putative membrane protein BlpL	CGSSpSV36_0032
bacteriocin locus	pncH	CGSSpSV36_0033
bacteriocin locus	putative immunity protein BlpX	CGSSpSV36_0034

**Table 3:** Subset of genes in SV35-T3 taken from genes unique to the parental strains as well as the antibiotic-resistance marker used to generate the strain.

Functional Category	Annotation	ID on parental genome
Spectinomycin-reistance gene	spectinomycin resistance gene	N/A
type 3 capsular locus from SV36-T3	transposase domain protein - **tnp**	CGSSpSV36_0284
type 3 capsular locus from SV36-T3	UDP-glucose 6-dehydrogenase (UDP-Glc dehydrogenase)(UDP-GlcDH) (UDPGDH) - **ugd**	CGSSpSV36_0285
type 3 capsular locus from SV36-T3	glycosyl transferase family 2 family protein - **wchE**	CGSSpSV36_0286
type 3 capsular locus from SV36-T3	hypothetical protein	CGSSpSV36_0289
phosphotransferase system (PTS) from SV35-T23	putative mannitol-specific phosphotransferase system (PTS), IIBC component	CGSSpSV35_0553
phosphotransferase system (PTS) from SV35-T23	putative transcriptional regulator	CGSSpSV35_0554
phosphotransferase system (PTS) from SV35-T23	putative mannitol-specific phosphotransferase system (PTS), IIA component	CGSSpSV35_0555
phosphotransferase system (PTS) from SV35-T23	mannitol-1-phosphate 5-dehydrogenase	CGSSpSV35_0556
phosphotransferase system (PTS) from SV35-T23	ABC-type polar amino acid transport system, ATPase component	CGSSpSV35_0557
bacteriocin locus from SV35-T23	pncR	CGSSpSV35_0662
bacteriocin locus from SV35-T23	pncT	CGSSpSV35_0663
bacteriocin locus from SV35-T23	bacteriocin blpM (or pncI)*	CGSSpSV35_0665
	hypothetical protein	CGSSpSV35_1729
	hypothetical protein	CGSSpSV35_1730

### Determinants of virulence in strains SV35-T23 and SV36-T3

Given these differences between SV35-T23 and SV36-T3, identification of determinants responsible for the superior virulence of strain SV36-T3 required additional experiments that involved construction of strain SV35-T3*: an SV35 recombinant that carried the type 3 capsular genes from strain SV36-T3.

The WT strains SV35-T23 and SV36-T3 along with strains SV36-T3*and SV35-T3* (both of which contained a spectinomycin resistance marker used in the construction of the type 3 transformants) were next evaluated for virulence using two different animal models: the murine intraperitoneal infection model and an adaptation of the chinchilla model of otitis media [27], [28].

### Evaluation of virulence using the murine intraperitoneal model

**Figure 4:**
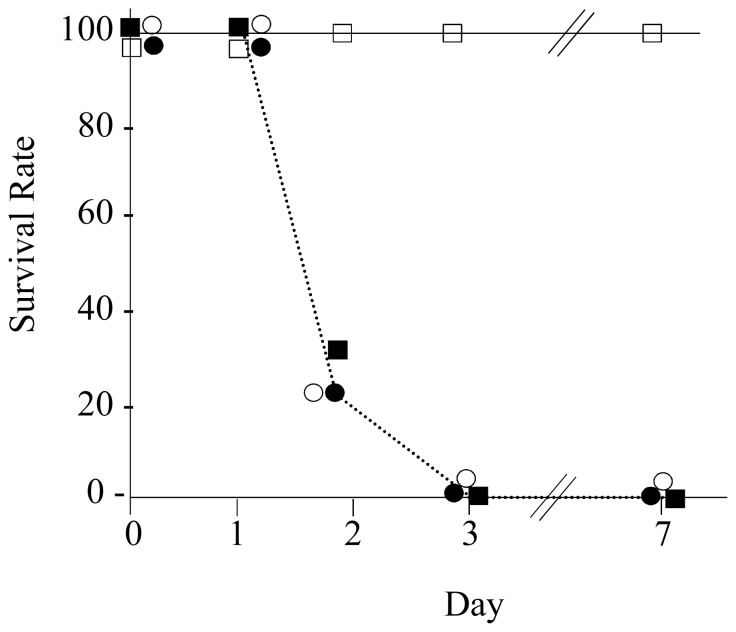
Mortality Comparison of the virulence of SV strains in the mouse intraperitoneal infection model. Bacterial strains grown in Todd-Hewitt broth were used to inoculate groups of mice (5 for each bacterial inoculum) – as described in the Methods. Strains expressing the type 3 capsular polysaccharide – SV36-T3 (○), SV36-T3* (carrying the spectinomycin resistance marker (•) and SV35-T3* (▪) – were each used at inoculum size of 10^3^ CFU per animal. Strain SV35-T23 (□) – expressing the capsular polysaccharide 23F – was used at inoculum size of 10^7^ CFU per animal. Survival rates were evaluated daily for 7 days – as described in the Methods.

The four strains tested in the mouse intraperitoneal model showed striking differences in their virulence which was related to the type of the capsular polysaccharide produced by the bacteria. Strains SV36-T3, SV36-T3* and SV35-T3* – each expressing a type 3 capsular polysaccharide – produced virtually superimposable survival curves: using 10^3^ CFU per animal for the i.p. injection, each one of these three strains killed 73 to 80% of animals two days after inoculation and all mice were killed three days after inoculation. In contrast, strain SV35-T23 expressing capsular polysaccharide 23F caused no lethal infection when injected into the animals at concentrations as high as 10^7^ CFU per animal even after seven days post inoculation (**Figure 4**).

### Evaluation of virulence using a chinchilla model

Otitis media (OM) caused by pneumococcal infections is one of the most frequent afflictions but it rarely develops into systemic disease and/or fatality in humans. A chinchilla model developed by Giebink and Quie [29] for the study of various features of pneumococcal infections causing middle ear disease provided important information concerning the modality of growth of bacterial strains in this anatomic space [30]–[32] and also about the mechanism of inflammation induced by the bacteria [33], [34]. More recently this model was modified and adapted to follow development of different degrees of otologic and systemic disease in the animals which could even lead to mortality. This version of the chinchilla model was used recently to compare the “pathogenic potential” of *S. pneumoniae* and *H. influenzae* clinical isolates [27], [28]. In spite of the fact that this model – unlike human otitis media – produces a fatal disease, we tested the SV35-T23 and SV36-T3 strains in this OM model since it provides a higher resolution in the rate of otologic and systemic disease, thus, possibly providing information on genetic determinants other than the capsule that may also contribute to the pathogenic potential of the SV36-T3 strain.

Using a transbullar procedure [27] four cohorts of at least nine young adult chinchillas each were inoculated bilaterally with wild type (WT) strains SV35-T23 and SV36-T3 as well as the *in vitro* generated recombinant strain SV35-T3* and the DNA donor strain SV36-T3*. The animals were evaluated daily for progression of systemic disease and each ear of each live animal was also evaluated daily for otologic (middle-ear) disease as well – rated on a severity scale of 0 to 4 with 0 being no disease and 4 representing a ruptured tympanic membrane (**Table 4**) [28]. Animals that died, or became moribund and had to be sacrificed, were given a systemic severity score of 5 for all time points following death.

**Table 4:** Multi-Parameter Scoring System to Quantify *Streptococcus Pneumoniae* Pathogenicity in the Chinchilla.

Otologic/systemic score	0	1	2	3	4	5
Status of tympanic membrane (TM)	Normal	Mild change	Moderate change	Frank purulence	TM rupture	
Systemic signs	None	Mild ataxia	Moderate ataxia, decreased oral intake	Lethargy, cornering, ocular discharge, fever	Moribund	Death

**Figure 5:**
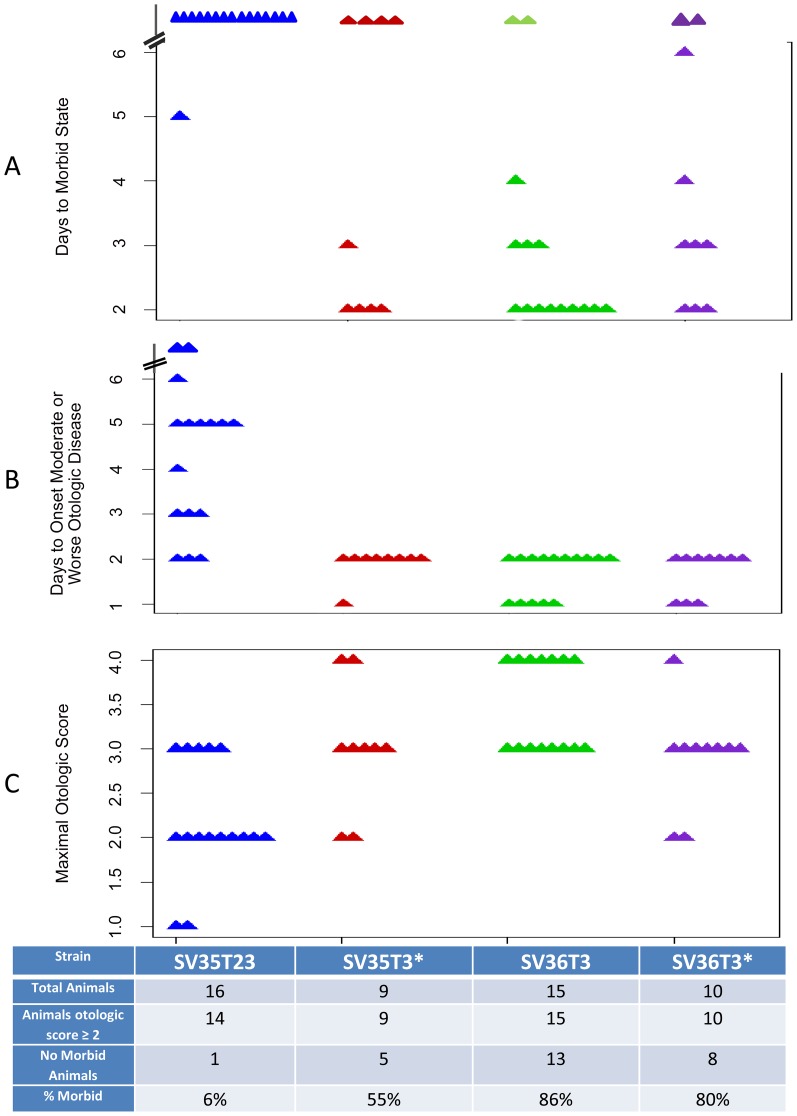
Phenotypic variation caused by a capsular switch from type 23 to type 3. Scatter plots illustrating differences in mortality and the outcome of otologic disease for animals infected with WT strainsSV35-T23 (blue symbols) and SV36-T3 (green symbols) and recombinant strains SV35-T3* (red symbols) and SV36-T3*(purple symbols) strains. Parameters are – as defined in Table 4– days to morbid state (A), days to onset of moderate or worst otologic disease (B), and maximal otologic score (C).

Data in Figure 5 show a comparison of the four strains – SV35-T23, SV35-T3*, SV36-T3 and SV36-T3* for the rate with which they acquired signs of systemic disease (morbid state) (Figure 5A); their rates of developing otologic disease (Figure 5B) and the number of animals showing maximal otologic score (Figure 5C) – as defined in Table 4.

In terms of reaching or surpassing a given otologic score, most animals inoculated with the three capsular type 3 strains have reached a score of ≥2. Specifically, 15 of the chinchillas inoculated with SV36-T3 had a maximal otologic score of 3 or more; 8 of the 10 chinchillas inoculated with SV36-T3* and 7 of the 9 animals that received the SV35-T3* inoculum also reached a score of 3 or 4. In contrast, only 5 of the 16 chinchillas inoculated with SV35-T23 reached an otologic score of 3 (Figure 5C).

The three groups of chinchillas that have received bacterial inocula expressing the type 3 capsule also showed close parallels in the days to onset of moderate or worse otologic disease which was reached rapidly within one or two days in these animals. This was in contrast to the case of the SV35-T23 inocula in which the days to onset of the otologic disease was spread between 2 to up to 6 or more days (Figure 5B).

All but one of the 16 animals inoculated with strain SV35-T23 survived while most animals inoculated with SV36-T3 died (13 of 15 animals or 86%). Mortality was also high among animals inoculated with SV36-T3* - i.e., bacteria expressing the type 3 capsule but also carrying the spectinomycin resistance marker: in this group 8 of the 10 animals (80%) died and 5 of the 9 animals (55%) inoculated with the recombinant strain SV35-T3* also died (Figure 5A).

Strains expressing the type 3 capsule showed superior virulence in both the mouse as well as the chinchilla model clearly identifying the capsule polysaccharide as the primary determinant of virulence.

Both capsule types 23F and 3 have been associated with high case fatality rates [35], [36]. In some studies, the type 3 capsule has been associated with a higher frequency of disease than any other serotype (including the 23F) [37]. Furthermore, the type 3 capsule is strongly associated with necrotizing pneumonia, as well as hemolytic uremic syndrome when compared to other serotypes [38], [39]. The type 3 is immediately distinguishable on blood agar plates where it forms larger colonies with a mucoid appearance.

In order to evaluate the possible significance of the smaller differences observed between strains SV36-T3 and SV35-T3* (i.e., the two strains expressing the same type 3 capsular polysaccharide), statistical evaluation of data was also done (Table 5).

**Table 5:** Probability values for differences in pathogenicity measures between the WT and recombinant *S. pneumoniae* strains.

	p-values							
Strain comparison	Day 1 Otoscopy Either ear	Day 2 Otoscopy Either ear	Rapidity of Developing Ear Fluid	Maximal Otologic Score	Time to Maximal Otologic Score	Overall Mortality@	Spread to Lung@	Spread to Brain@
SV35-T23 vs. SV36-T3	***0.0008***	***0.0002*** *	***0.0019*** *	***0.0019*** *	NC	1.1^−05^	***0.0058***	***0.023***
SV35-T3* vs. SV36-T3	***0.0387***	***0.0031*** *	0.298*	0.1806*	NC	0.0823	0.3348	1

Italicized values are statistically significant at P≤0.05.

* computed using the Mann-Whitney U (Wilcox) Test;

@ computed using the Fisher Exact Test for Count Data, NC = not computed.

The mean systemic severity scores calculated for the cohorts of animals infected with SV35-T3* and SV36-T3 for day 1 were 0.39 and 1.05 respectively. The mean severity scores for the same cohorts on day 2 were 2.28 and 3.22 respectively, i.e., the average severity score for the SV35-T3* infected animals were nearly a full point lower than the score obtained for SV36-T3 cohort. The difference in local middle ear disease (as determined by otoscopy) was statistically significant with a p value of 0.0387 on day 1 and 0.0031 on day 2 – computed using the Mann-Whitney (Wilcoxon) test. However, there was no statistical significance between the two strains with respect to other parameters (e.g., maximal otologic scores, rapidity of ear fluid development).

These data raise the interesting possibility that some as yet unidentified factors in addition to the capsular type 3 may also contribute to the capacity of these strains to cause OM disease in the chinchilla model. Confirmation of this observation and identification of the hypothetical determinants will require additional studies. The effect of genetic background on the virulence potential of capsular type 3 transformants generated in the laboratory has been described in several *S. pneumoniae* isolates [40].

## Supporting Information

Table S1 **PCR Primers Used for Capsule Switch.**
(DOC)

## References

[1] Aniansson G, Alm B, Andersson B, Larsson P, Nylén O, et al (1992) Nasopharyngeal colonization during the first year of life. J Infect Dis 165 Suppl 1: S38–42. 1588174 10.1093/infdis/165-supplement_1-s38

[2] Austrian R (1986) Some aspects of the pneumococcal carrier state. J Antimicrob Chemother 18 Suppl A: 35–45. 10.1093/jac/18.supplement_a.35 3745031

[3] Gray BM, Converse GM, Dillon HC (1980) Epidemiologic studies of *Streptococcus pneumoniae* in infants: acquisition, carriage, and infection during the first 24 months of life. J Infect Dis 142: 923–933. 7462701 10.1093/infdis/142.6.923

[4] Bogaert D, De Groot R, Hermans PW (2004) *Streptococcus pneumoniae* colonisation: the key to pneumococcal disease. Lancet Infect Dis 4: 144–154. 14998500 10.1016/S1473-3099(04)00938-7

[5] World Health Organizaiton website. *Streptococcus pneumoniae* 2008; Available: http://www.who.int/vaccine_research/diseases/ari/en/index5.html. Accessed 2012 September 5.

[6] Rioseco ML, Riquelme R (2004) Bacteremic pneumococcal pneumonia in 45 immunocompromised hospitalized adults. Rev Med Chil 132: 588–594. 15279145 10.4067/s0034-98872004000500008

[7] Zimmer C, Beiderlinden M, Peters J (2006) Lethal acute respiratory distress syndrome during anti-TNF-alpha therapy for rheumatoid arthritis. Clin Rheumatol 25: 430–432. 16200383 10.1007/s10067-005-0008-1

[8] Gates GA (1996) Cost-effectiveness considerations in otitis media treatment. Otolaryngol Head Neck Surg 114: 525–530. 8643261 10.1016/S0194-59989670243-7

[9] Pichichero ME, Casey JR (2007) Evolving microbiology and molecular epidemiology of acute otitis media in the pneumococcal conjugate vaccine era. Pediatr Infect Dis J 26 10 Suppl S12–6. 18049375 10.1097/INF.0b013e318154b25d

[10] Daly KA, Hoffman HJ, Kvaerner KJ, Kvestad E, Casselbrant ML, et al (2009) Epidemiology, natural history, and risk factors: Panel report from the Ninth International Research Conference on Otitis Media. Int J Pediatr Otorhinolaryngol 74: 231–240. 19836843 10.1016/j.ijporl.2009.09.006

[11] Mitchell TJ (2004) Pneumolysin and Other Virulence Proteins. In The Pneumococcus, ETuomanen, Editor. 2004, ASM Press: Washington, D.C. pp. 61–74.

[12] Kamerling JP (2000) Pneumococcal polysaccharides: A chemical view. In *Streptococccus pneumoniae*. Moleculary Biology & Mechanisms of Disease, ATomasz, Editor. Mary Ann Liebert, Inc. Larchmont, NY. pp. 81–114.

[13] Bentley SD, Aanensen DM, Mavroidi A, Saunders D, Rabbinowitsch E, et al (2006) Genetic analysis of the capsular biosynthetic locus from all 90 pneumococcal serotypes. PLoS Genet 2: e31. 16532061 10.1371/journal.pgen.0020031 PMC1391919

[14] Weinberger DM, Harboe ZB, Sanders EA, Ndiritu M, Klugman KP, et al (2010) Association of serotype with risk of death due to pneumococcal pneumonia: a meta-analysis. Clin Infect Dis 51: 692–699. 20715907 10.1086/655828 PMC2927802

[15] Briles DE, Crain MJ, Gray BM, Forman C, Yother J (1992) Strong association between capsular type and virulence for mice among human isolates of *Streptococcus pneumoniae* . Infect Immun 60: 111–116. 1729176 10.1128/iai.60.1.111-116.1992 PMC257510

[16] Croucher NJ, Harris SR, Fraser C, Quail MA, Burton J, et al (2011) Rapid pneumococcal evolution in response to clinical interventions. Science 331: 430–434. 21273480 10.1126/science.1198545 PMC3648787

[17] Hiller NL, Ahmed A, Powell E, Martin DP, Eutsey R, et al (2010) Generation of genic diversity among *Streptococcus pneumoniae* strains via horizontal gene transfer during a chronic polyclonal pediatric infection. PLoS Pathog 16;6pii: e1001108. 10.1371/journal.ppat.1001108 PMC2940740 20862314

[18] Sá-Leão R, Tomasz A, Santos Sanches I, de Lencastre H (2002) Pilot study of the genetic diversity of the pneumococcal nasopharyngeal flora among children attending day care centers. J Clin Microbiol 40: 3577–3585. 12354849 10.1128/JCM.40.10.3577-3585.2002 PMC130868

[19] Carter RJ, Sorenson G, Heffernan R, Kiehlbauch JA, Kornblum JS, et al (2005) MDRSP Working Group. Failure to control an outbreak of multidrug-resistant *Streptococcus pneumoniae* in a long-term-care facility: emergence and ongoing transmission of a fluoroquinolone-resistant strain. Infect Control Hosp Epidemiol 26: 248–255. 15796275 10.1086/502534

[20] Perrone C, Perrone P, Kopetz V, Nedunchezian D, Leggiadro R (2000) Prevalence of penicillin-nonsusceptible pneumococcal bacteremia in a Staten Island community hospital. South Med J 93: 1078–1080. 11095556

[21] Nesin M, Ramirez M, Tomasz A (1998) Capsular transformation of a multidrug-resistant *Streptococcus pneumoniae* in vivo. J Infect Dis 177: 707–713. 9498451 10.1086/514242

[22] Hiller NL, Eutsey RA, Powell E, Earl J, Janto B, et al (2011) Comparative Genomics of Phenotypically Diverse Clinical Pandemic Multidrug-Resistant *Streptococcus pneumoniae* Strains from the PMEN1 lineage. PLoS ONE 6: e28850. 22205975 10.1371/journal.pone.0028850 PMC3242761

[23] Darling AC, Mau B, Blattner FR, Perna NT (2004) Mauve: multiple alignment of conserved genomic sequence with rearrangements. Genome Res 14: 1394–1403. 15231754 10.1101/gr.2289704 PMC442156

[24] Ehrlich GD, Ahmed A, Earl J, Hiller NL, Costerton JW, et al (2010) The Distributed Genome Hypothesis as a Rubric for Understanding Evolution in situ During Chronic Bacterial Biofilm Infectious Processes. FEMS Immunol Med Microbiol 59: 269–279. 20618850 10.1111/j.1574-695X.2010.00704.x PMC2910629

[25] Ehrlich GD, Hu FZ, Shen K, Stoodley P, Post JC (2005) Bacterial Plurality as a General Mechanism Driving Persistence in Chronic Infections. Clin Orthop Relat Res 437: 20–24. 10.1097/00003086-200508000-00005 PMC1351326 16056021

[26] Forbes ML, Horsey E, Hiller NL Buchinsky FJ, Hayes JD, et al (2008) Strain-specific Virulence Phenotypes of *Streptococcus pneumoniae* Assessed Using the *Chinchilla laniger* Model of Otitis Media. PLoS One 3 4 e1969 doi:10.1371/journal.pone.0001969. 18398481 10.1371/journal.pone.0001969 PMC2279396

[27] Krzywinski MI, Schein JE, Birol I, Connors J, Gascoyne R, et al (2009) Circos: An information aesthetic for comparative genomics. Genome Res Doi:10.1101/gr.092759.109. 10.1101/gr.092759.109 PMC2752132 19541911

[28] Buchinsky FJ, Forbes ML, Hayes JD, Shen K, Ezzo S, et al (2007) Virulence phenotypes of low-passage clinical isolates of nontypeable *Haemophilus influenzae* assessed using the chinchilla laniger model of otitis media. BMC Microbiol 7: 56. 17570853 10.1186/1471-2180-7-56 PMC1914350

[29] Giebink GS, Payne EE, Mills EL, Juhn SK, Quie PG (1976) Experimental otitis media due to *Streptococcus pneumoniae*: immunopathogenic response in the chinchilla. J Infect Dis 134: 595–604. 12236 10.1093/infdis/134.6.595

[30] Bakaletz LO (2009) Chinchilla as a robust, reproducible and polymicrobial model of otitis media and its prevention. Expert Rev Vaccines 8: 1063–1082. 19627188 10.1586/erv.09.63

[31] Apicella MA (2009) Bacterial otitis media, the chinchilla middle ear, and biofilms. J Infect Dis 199 6 774–775. 19434910 10.1086/597043

[32] Ehrlich GD, Veeh R, Wang X, Costerton JW, Hayes JD, et al (2002) Mucosal biofilm formation on middle-ear mucosa in the chinchilla model of otitis media. JAMA 287: 1710–1715. 11926896 10.1001/jama.287.13.1710

[33] Post JC, Ehrlich GD (2009) Biofilms and their role in ear and respiratory infections. In: Snow JB, Wackym PA, editors. Otorhinolaryngology Head and Neck Surgery, 17th Edition. Hamilton, Ontario: BC Decker Publisher p. 839–845.

[34] Carlsen BD, Kawana M, Kawana C, Tomasz A, Giebink GS (1992) Role of the bacterial cell wall in middle ear inflammation caused by *Streptococcus pneumoniae* . Infect Immun 60(: 2850–2854. 1612750 10.1128/iai.60.7.2850-2854.1992 PMC257244

[35] Ruckinger S, von Kries R, Siedler A, van der Linden M (2009) Association of serotype of *Streptococcus pneumoniae* with risk of severe and fatal outcome. Pediatr Infect Dis J 28: 118–122. 19116604 10.1097/INF.0b013e318187e215

[36] Hausdorff WP, Bryant J, Paradiso PR, Siber GR (2000) Which pneumococcal serogroups cause the most invasive disease: implications for conjugate vaccine formulation and use, part I. Clin Infect Dis 30: 100–121. 10619740 10.1086/313608

[37] Henriques B, Kalin M, Ortqvist A, Liljequist B, Almela M, et al (2000) Molecular epidemiology of pneumococcal pneumonia causing invasive disease in 5 countries. J Infect Dis 182: 833–839. 10950778 10.1086/315761

[38] Bender JM, Ampofo K, Korgenski K, Daly J, Pavia AT, et al (2008) Pneumococcal necrotizing pneumonia in Utah: does serotype matter? Clin Infect Dis. 46:1346–1352. Erratum in: Clin Infect Dis 47: 437. 10.1086/586747 PMC3673544 18419434

[39] Bender JM, Ampofo K, Byington CL, Grinsell M, Korgenski K, et al (2010) Epidemiology of *Streptococcus pneumoniae*-induced hemolytic uremic syndrome in Utah children. Pediatr Infect Dis J29: 712–716. 10.1097/INF.0b013e3181db03a7 20661100

[40] Kelly T, Dillard JP, Yother J (1994) Effect of genetic switching of capsular type on virulence of *Streptococcus pneumoniae* . Infect Immun 62 5 1813–1819. 8168944 10.1128/iai.62.5.1813-1819.1994 PMC186414

