# Supplementary material for: In Vivo Capsular Switch in Streptococcus pneumoniae – Analysis by Whole Genome Sequencing
Source: PLoS One. 2012 Nov 8;7(11):e47983. doi: 10.1371/journal.pone.0047983 (PMC3493582; doi:10.1371/journal.pone.0047983)
Supplement: Table S1 — PCR Primers Used for Capsule Switch. (DOC) [file pone.0047983.s001.doc]

Table S1 **PCR Primers Used for Capsule Switch**

| Oligo# | Name | Sequence | Product |
| --- | --- | --- | --- |
| 12921 | tnp1 amp region 1F | ATTGCAGAAGCTAAGAAGCG | Amplifies transposase SV36-T3_312 (tnp1) and flanking regions |
| 12922 | tnp1 amp region 1R | CTGCTTTAACTACAGCTAAACTACT |  |
|  |  |  |  |
| 12956 | pGEMT tnp1F XmaI | ATATCCCGGG GAGGAAGAGGTAAAAGTTTA | Inverse PCR primers amplifying pGEMT and tnp1 with XmaI and BamHI |
| 12957 | pGEMT tnp1R BamHI | ATATGGATCC GAAAACATGGGCTCATATCA | restriction sites |
|  |  |  |  |
| 13021 | Spec Forward BamHI | ATATGGATCC TCCCCCGTTTGATTTTTAATGGTAATGTGATAAA | Amplifies Spec cassette from plasmid pr412 with BamHI and XmaI |
| 13022 | Spec Reverse XmaI | ATATCCCGGG CGGAATGGATCCAATTTTTTTA | restriction sites |
|  |  |  |  |
| 13044 | Spec tnp1 22.0 F | CCAGTATCGCAAATGGGAGCTTCA | Amplifies SV36-T3 containing tnp1 spec and flanking regions |
| 13045 | Spec tnp1 22.0 R | TTGTGGGCGTAAGCCTTAACATAG |  |
|  |  |  |  |
| 13106 | SV36-T3 Spec Capsule Part 1F | GCTTGATTTTGTTGTCAGGTTCAT | upstream flank of type 3 capsule region (Forward) into capsule region (Reverse) |
| 13107 | SV36-T3 Spec Capsule Part 1R | TCAACTCCTGATCCAAACATGTAA |  |
| 13108 | SV36-T3 Spec Capsule Part 2F | GGTGCTTTTTGATATGAGCCCATG | section of type 3 capsule region including tnp1 spec |
| 13109 | SV36-T3 Spec Capsule Part 2R | AATTCCCTCCACTTTCCAACTGAG |  |
| 13110 | SV36-T3 Spec Capsule Part 3F | ACTCCCTAGCAGATGGCGATCGTG | section of type 3 capsule |
| 13111 | SV36-T3 Spec Capsule Part 3R | CAACACATTCTCTACCGCACACGC |  |
| 13112 | SV36-T3 Spec Capsule Part 4F | CAATTGACGGCTTGAGCTCCAATC | section of type 3 capsule |
| 13113 | SV36-T3 Spec Capsule Part 4R | TCGTTTTTTGGGCCGTTAATAACC |  |
| 13114 | SV36-T3 Spec Capsule Part 5F | TCTTATTCGTTGGGCGGTTATATA | section of type 3 capsule |
| 13115 | SV36-T3 Spec Capsule Part 5R | GGTTTGTGTAAAAGGCGTCTTCTT |  |
| 13116 | SV36-T3 Spec Capsule Part 6F | AATTACGTTATTGCACTTGGTAAGC | downstream flank of type 3 capsule region(Reverse) starting in capsule region(Forward) |
| 13117 | SV36-T3 Spec Capsule Part 6R | GTGCAATGCTTAACCCTGATAAAT |  |
